# miRNA Studies in Glaucoma: A Comprehensive Review of Current Knowledge and Future Perspectives

**DOI:** 10.3390/ijms241914699

**Published:** 2023-09-28

**Authors:** Margarita Dobrzycka, Anetta Sulewska, Przemyslaw Biecek, Radoslaw Charkiewicz, Piotr Karabowicz, Angelika Charkiewicz, Kinga Golaszewska, Patrycja Milewska, Anna Michalska-Falkowska, Karolina Nowak, Jacek Niklinski, Joanna Konopińska

**Affiliations:** 1 Department of Ophthalmology, Medical University of Bialystok, 15-276 Bialystok, Poland; margaritakuratczyk@gmail.com (M.D.); kin.golaszewska@gmail.com (K.G.); 2 Department of Clinical Molecular Biology, Medical University of Bialystok, 15-269 Bialystok, Poland; anetta.sulewska@umb.edu.pl (A.S.); angelika.charkiewicz@umb.edu.pl (A.C.); jacek.niklinski@umb.edu.pl (J.N.); 3 Faculty of Mathematics and Information Science, Warsaw University of Technology, 00-662 Warsaw, Poland; przemyslaw.biecek@pw.edu.pl; 4 Center of Experimental Medicine, Medical University of Bialystok, 15-369 Bialystok, Poland; radoslaw.charkiewicz@umb.edu.pl; 5 Biobank, Medical University of Bialystok, 15-269 Bialystok, Poland; piotr.karabowicz@umb.edu.pl (P.K.); patrycja.milewska@umb.edu.pl (P.M.); anna.michalska-falkowska@umb.edu.pl (A.M.-F.); 6 Department of Obstetrics and Gynecology, C.S. Mott Center for Human Growth and Development, School of Medicine, Wayne State University, Detroit, MI 48201, USA; karolinanowak@wayne.edu

**Keywords:** glaucoma, miRNA, liquid biopsy, personalized medicine, next-generation sequencing

## Abstract

Glaucoma, a neurodegenerative disorder that leads to irreversible blindness, remains a challenge because of its complex nature. MicroRNAs (miRNAs) are crucial regulators of gene expression and are associated with glaucoma and other diseases. We aimed to review and discuss the advantages and disadvantages of miRNA-focused molecular studies in glaucoma through discussing their potential as biomarkers for early detection and diagnosis; offering insights into molecular pathways and mechanisms; and discussing their potential utility with respect to personalized medicine, their therapeutic potential, and non-invasive monitoring. Limitations, such as variability, small sample sizes, sample specificity, and limited accessibility to ocular tissues, are also addressed, underscoring the need for robust protocols and collaboration. Reproducibility and validation are crucial to establish the credibility of miRNA research findings, and the integration of bioinformatics tools for miRNA database creation is a valuable component of a comprehensive approach to investigate miRNA aberrations in patients with glaucoma. Overall, miRNA research in glaucoma has provided significant insights into the molecular mechanisms of the disease, offering potential biomarkers, diagnostic tools, and therapeutic targets. However, addressing challenges such as variability and limited tissue accessibility is essential, and further investigations and validation will contribute to a deeper understanding of the functional significance of miRNAs in glaucoma.

## 1. Introduction

Glaucoma is a complex and heterogeneous eye disorder that poses significant health challenges globally and is characterized by elevated intraocular pressure (IOP), leading to the degeneration of retinal ganglion cells (RGCs) and their axons [1]. Based on the anatomy of the anterior chamber angle, glaucoma encompasses various subtypes, including primary open-angle glaucoma (POAG), primary angle-closure glaucoma, normal-tension glaucoma (NTG), and pseudoexfoliation glaucoma (PEXG). However, conventional diagnostic and therapeutic methods often fail to provide precise and personalized care for patients [1,2]. Moreover, the mechanisms underlying glaucoma pathogenesis are not fully understood, although they are influenced by genetic and environmental factors [2]. The field of microRNA (miRNA) research offers a promising avenue to elucidate the intricate molecular mechanisms underlying glaucoma [3].

MicroRNAs (miRNAs) are small non-coding ribonucleic acid (RNA) molecules that play a crucial role in post-transcriptional gene regulation and influence various biological processes, including cellular growth, differentiation, development, and apoptosis. These endogenous RNA molecules typically consist of approximately 22 nucleotides and regulate gene expression by binding to complementary mRNA sequences, resulting in mRNA degradation or translational repression. Encoded within the genome, miRNAs undergo a complex maturation process involving cleavage events mediated by the ribonuclease III enzymes Drosha and Dicer, ultimately generating mature miRNAs. miRNAs are highly conserved across species, highlighting their evolutionary and functional importance [4,5].

Studies have revealed altered expression patterns of miRNAs in various tissues and body fluids of both glaucoma patients and animal models [3,6]. Dysregulated miRNAs can modulate the critical pathways and processes involved in glaucoma, including apoptosis, autophagy, neurogenesis, aging, extracellular matrix remodeling, oxidative stress, inflammation, and angiogenesis [3]. Moreover, certain miRNAs show promise as non-invasive diagnostic or prognostic biomarkers because they can be easily detected in blood or tear samples [3]. Specific miRNAs have emerged as promising therapeutic targets or agents for glaucoma treatment because their activities can be manipulated using synthetic mimics or inhibitors [3].

In this comprehensive review, we aim to thoroughly explore miRNA studies in the context of glaucoma. We delved into the intricacies of these molecular investigations focusing on miRNAs and meticulously examined their advantages and disadvantages. This encompasses the potential of miRNAs to serve as early detection and diagnostic biomarkers, their capacity to illuminate molecular pathways and mechanisms, their implications for personalized medicine, therapeutic possibilities, and their application in non-invasive monitoring strategies. Furthermore, we address the constraints inherent in miRNA research concerning glaucoma, including factors such as variability, limited sample sizes, sample specificity, and the complexities arising from restricted access to ocular samples. To address these challenges effectively, we emphasize the significance of implementing robust research protocols and fostering collaborative efforts. We also highlight the credibility of the miRNA research outcomes and the importance of reproducibility and rigorous validation. Additionally, we underscore the value of incorporating bioinformatic tools in the establishment of miRNA databases, which serve as a foundational component for comprehensive approaches that can be used to investigate miRNA dysregulation in patients with glaucoma.

## 2. MiRNA Biogenesis and Silencing Mechanisms

Understanding the biogenesis and silencing mechanisms of miRNAs is crucial in deciphering their roles in glaucoma. MicroRNA biogenesis is a highly regulated and intricate process that involves several steps—from transcription to the generation of mature, functional miRNAs. This process plays a pivotal role in post-transcriptional gene regulation and is crucial for fine-tuning gene expression in various biological processes, including development, differentiation, and disease pathogenesis [5].

**Figure 1:**
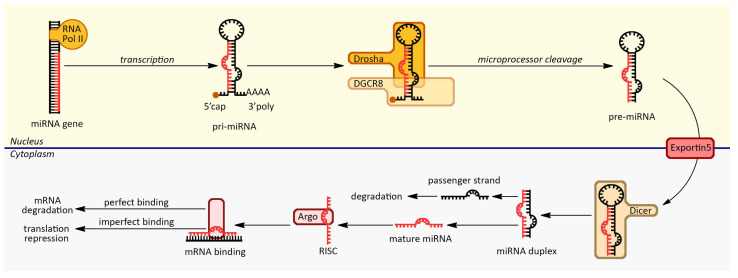
miRNA biogenesis and the silencing mechanism.

MiRNA genes, typically found in the genomes of most organisms, are transcribed by RNA polymerase II or III into primary miRNA transcripts (pri-miRNAs). These pri-miRNAs can be located within intronic regions of protein-coding genes or in intergenic regions. The next step in miRNA biogenesis is the processing of the pri-miRNA in the nucleus. This processing is carried out by a protein complex known as the microprocessor complex, which includes the RNase III enzyme Drosha and its cofactor, DGCR8. The microprocessor complex cleaves the pri-miRNA to produce a hairpin-shaped precursor miRNA (pre-miRNA) that is typically around 70 nucleotides long. Once formed, the pre-miRNA is exported from the nucleus to the cytoplasm by the exportin-5 protein, a RanGTP-dependent transporter. In the cytoplasm, the pre-miRNA undergoes further processing by the RNase III enzyme Dicer, which cleaves it into a shorter RNA duplex. This duplex consists of the mature miRNA (guide strand) and its complementary strand (passenger strand). The mature miRNA is then incorporated into the RNA-Induced Silencing Complex (RISC), which is composed of Argonaute proteins and other associated factors. Within the RISC, the guide strand of the miRNA directs the complex to target mRNAs with complementary sequences. The primary function of mature miRNAs is to silence or regulate gene expression post-transcriptionally. They achieve this by binding to the 3′ untranslated region (UTR) of target mRNAs through partial base pairing between the miRNA’s “seed sequence” and complementary sequences in the mRNA. This interaction leads to gene silencing through two primary mechanisms: (1) mRNA degradation, which encompasses perfect or near-perfect complementarity between the miRNA and the target mRNA, which can trigger mRNA degradation. In this scenario, the target mRNA is cleaved by the Ago protein within the RISC complex, leading to its degradation and decreased protein production; (2) translation repression, when miRNA-mRNA binding is imperfect, as is often the case, the miRNA-RISC complex can inhibit translation initiation and elongation. This results in reduced protein synthesis from the target mRNA without mRNA degradation. It is important to note that miRNAs can regulate multiple target mRNAs simultaneously, and a single mRNA can be targeted by multiple miRNAs, adding a layer of complexity to the regulatory network [5,6] (Figure 1).

As elucidated earlier, it becomes evident that miRNA biogenesis and silencing mechanisms serve as critical components within the realm of post-transcriptional gene regulation. The dysregulation of these processes can have profound implications in various diseases, including those with a genetic basis such as glaucoma, making them attractive targets for therapeutic interventions and further exploration in the context of this blinding condition.

## 3. miRNA Profiling Tools in Glaucoma

Regarding the captivating realm of miRNA research in the context of glaucoma, it is essential to delve into the intricacies of the tools employed in these scientific endeavors. miRNA profiling, a cornerstone of such investigations, encompasses a rich repertoire of distinct techniques, with each offering unique advantages and limitations.

### 3.1. Real-Time Polymerase Chain Reaction (RT-PCR)

This method uses stem-looped primers or specific oligonucleotides to extend and reverse transcribe miRNAs, followed by amplification and quantification using miRNA-specific primers or probes. It offers simplicity, convenience, and cost-effectiveness but has limited throughput, requires reference genes and duplicates, and provides relative quantification [7,8,9].

### 3.2. Digital Droplet PCR (ddPCR)

This technique employs water–oil emulsion droplet technology to perform a digital polymerase chain reaction (PCR) within each droplet based on the Poisson distribution. This enables the determination of absolute copy numbers of miRNAs without the need for a standard curve. ddPCR demonstrates exceptional precision and high sensitivity and provides absolute quantification, eliminating the need for duplicates or reference genes. However, it is relatively expensive, requires a specific system, and has limited throughput [10,11,12].

### 3.3. miRNA Arrays

Fluorescently labeled miRNAs hybridize to complementary probes immobilized on glass slides. The resulting fluorescence signals are scanned and quantified to determine the expression levels of specific miRNAs. miRNA arrays allow for the simultaneous profiling of thousands of miRNAs, offering cost-effectiveness but demanding substantial RNA input. Moreover, they exhibit reduced sensitivity, provide relative quantification, have a restricted dynamic range, and are limited to known miRNAs [13,14].

### 3.4. NanoString nCounter-Based miRNA Assays

This method employs molecular barcodes to detect individual miRNAs without the need for reverse transcription or amplification. Each miRNA is ligated to an miR-tag and paired with a capture and reporter probe complex. These complexes are immobilized on a cartridge, and the unique barcodes are subsequently counted. The NanoString nCounter-based method allows for rapid and straightforward sample preparation and data analysis. However, this approach is limited to known miRNAs, requires access to a specific platform, and incurs high costs per run [15,16].

### 3.5. Small RNA Sequencing

This technique involves the ligation and reverse transcription of miRNAs using 5’ and 3’ adaptors, followed by high-throughput sequencing and bioinformatics analysis. It covers a wide range of small RNA species, including novel sequences, requires minimal RNA input, and exhibits remarkable sensitivity and a broad dynamic range. However, it generates substantial amounts of data that require computational expertise and incurs relatively high costs [17,18].

### 3.6. miRNA Luciferase Reporter Assay

**Figure 2:**
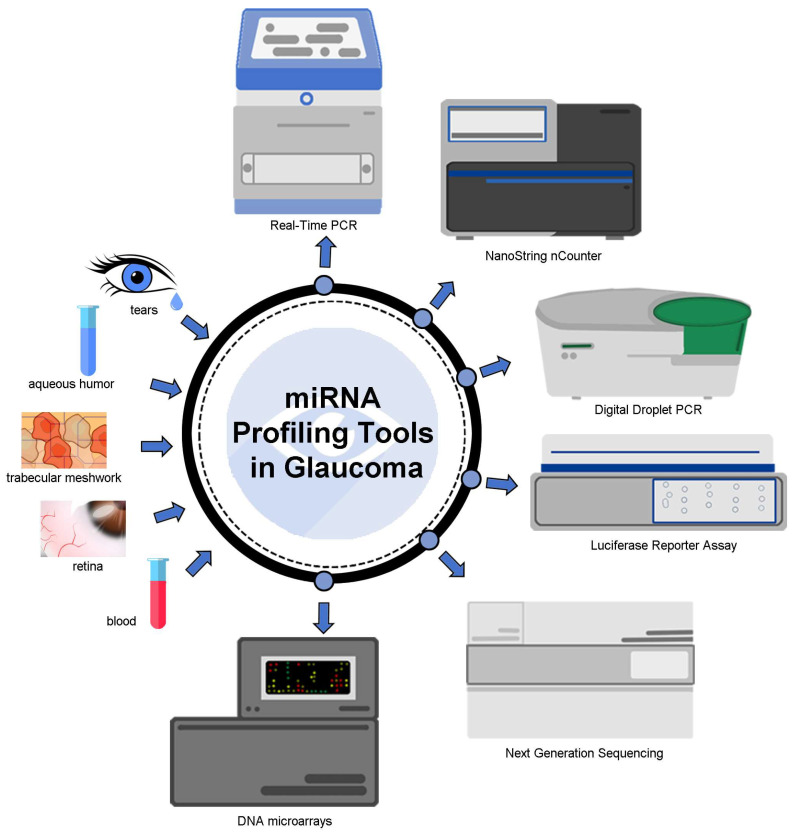
miRNA profiling tools in glaucoma.

This method necessitates a construct that combines the luciferase gene with the 3′ UTR of the target gene for the anticipated miRNA. The construct is then transfected into the cells and the miRNA mimic of interest. The resulting luciferase activity is measured to determine the interaction between the miRNA and the target gene. The miRNA luciferase reporter assay is an economical and straightforward approach that can be performed in live cells. However, it requires miRNA mimic transfection, which may induce cytotoxicity, and is not conducted using disease-relevant tissues or cells [19,20]. A graphical representation of miRNA profiling tools is showcased in Figure 2.

MiRNA profiling tools offer significant potential in the routine management of glaucoma patients by enabling early detection, personalized treatment plans, and the continuous monitoring of disease progression and treatment response. These tools can identify specific miRNA signatures associated with glaucoma, helping with early diagnosis and risk stratification. They also inform personalized treatment decisions, potentially uncover novel therapeutic targets, and provide prognostic information. By analyzing miRNA profiles, clinicians can tailor interventions, track treatment effectiveness, and improve patient outcomes. However, further research and validation are needed to fully integrate miRNA profiling into clinical practice for glaucoma management [7,21].

## 4. Advantages of miRNA in Glaucoma

Understanding the advantages and limitations of miRNA research in glaucoma is essential to evaluate its potential as a diagnostic tool, therapeutic target, and non-invasive monitoring approach. By examining both the benefits and challenges, researchers can effectively harness the power of miRNA research to advance glaucoma management and personalized medical approaches.

### 4.1. Biomarkers for Early Detection and Diagnosis

miRNA research in the context of glaucoma offers a key advantage through the potential use of miRNAs as biomarkers for the diagnosis, prognosis, and prediction of diseases [7,21]. Biomarkers are measurable indicators that provide valuable information for clinical decision-making. MiRNAs possess numerous attributes that make them promising candidates for biomarkers, including stability, specificity, accessibility, and modifiability [22].

#### 4.1.1. miRNA Stability

miRNAs are highly stable molecules that can withstand degradation by nucleases and extreme conditions such as high temperatures (80 °C), low pH, and freeze–thaw cycles [23,24,25]. This exceptional stability makes miRNAs suitable as biomarkers and therapeutic agents for various diseases, including cancer, cardiovascular diseases, and neurodegenerative disorders [26,27,28].

The stability of miRNAs can be attributed to several factors. First, their short length (approximately 22 nucleotides) contributes to their resistance to degradation and facilitates their persistence in various biological fluids and tissues [29,30]. Second, miRNAs associate with RNA-binding proteins such as Argonaute and Dicer, as well as ribonucleoprotein complexes such as microprocessors and RISC, providing protection against enzymatic degradation [31]. Third, miRNAs can be encapsulated within extracellular vesicles and exosomes, shielding them from degradation and facilitating their transport to target cells [32]. Finally, miRNAs can undergo chemical modifications such as methylation or phosphorylation, which prevent recognition and cleavage by nucleases and enhance miRNA stability [33,34].

#### 4.1.2. Sources of miRNAs for Glaucoma Diagnosis

The stability of miRNAs enables their reliable detection and measurement in various biological samples, including the aqueous humor (AH), tears, trabecular meshwork (TM), retina, and blood [3]. This stability also opens up new avenues for the development of non-invasive diagnostic tests wherein miRNAs can be easily detected and quantified.

AH, which fills the anterior chamber of the eye and maintains IOP, serves as an informative biofluid that reflects the physiological and pathological conditions of ocular tissues, including miRNA expression [35,36]. AH contains a diverse array of cells and molecules, including cytokines, growth factors, prostaglandins, and miRNAs, all of which contribute to ocular homeostasis and immunity [37,38,39,40]. The collection of AH samples offers stability and resistance to degradation by nucleases and environmental factors [41]. However, it is important to consider the various factors that can influence the stability of AH samples, including the collection method, storage conditions, extraction protocols, and quantification techniques. Sterile syringes or needles are recommended for contamination-free collection to ensure the integrity of miRNAs in AH samples [42,43,44,45].

Tears, secreted by the lacrimal glands, contribute to ocular surface lubrication and serve as a viable source of miRNAs. These biofluids contain a diverse range of cells and molecules, including miRNAs involved in ocular homeostasis and immunity [7,46]. Tears offer a non-invasive approach to assess changes in the ocular surface, making them valuable for the diagnosis and monitoring of glaucoma. However, the stability of tear samples can be influenced by factors such as volume, flow rate, film stability, ocular surface inflammation, and potential for contamination by other fluids or substances. To ensure the integrity of miRNAs in tear samples, sterile swabs or Schirmer strips should be used to minimize stimulation and avoid reflex tearing or irritation [47].

TM is an important source of miRNAs because of its critical role in regulating AH outflow and maintaining IOP [48,49]. Comprising cells and an extracellular matrix, the TM forms a porous structure that enables the drainage of the AH from the anterior chamber to Schlemm’s canal [50]. Within the TM, a wide range of miRNAs that influence key aspects of glaucoma pathogenesis and progression, including IOP regulation, oxidative stress, inflammation, extracellular matrix remodeling, and angiogenesis, are expressed. These miRNAs modulate TM function and structure [7,49,51]. TM samples are stable and can be obtained from TM tissues or cells using various methods such as laser capture microdissection (LCM), cell culture, or tissue homogenization [52,53]. However, the stability of TM samples depends on factors such as sample quality and quantity, preservation methods, extraction protocols, and quantification techniques. To ensure optimal miRNA integrity, TM samples should be promptly collected from fresh or frozen donor eyes or surgical specimens to minimize delays that may lead to tissue degradation or RNA loss [54].

The retina, located in the posterior part of the eye, is a valuable source of miRNAs [55]. This light-sensitive tissue consists of photoreceptors and RGCs which play crucial roles in converting light signals into neural impulses that are transmitted through the optic nerve to the brain. Glaucoma results in the progressive degeneration of RGCs and their axons within the retina [56,57]. The retina expresses various miRNAs that govern essential processes such as RGC survival, axonal transport, synaptic transmission, and neuroinflammation [55]. Retinal samples are stable and can be extracted from retinal tissues or cells using diverse methods such as LCM, cell culture, and tissue homogenization [58,59,60,61]. However, the stability of these samples may be influenced by factors such as the type and stage of glaucoma; location and size of the retinal sample; and the preservation method, extraction protocol, and quantification technique employed. To guarantee the utmost preservation of miRNAs, it is essential to promptly gather retinal samples from recently obtained or cryopreserved donor eyes or surgical specimens, with the aim of minimizing tissue deterioration or RNA depletion [62,63,64,65,66].

Blood, a valuable source for miRNA collection, is a circulating biofluid containing diverse cells and molecules which can be categorized into plasma and serum, each with distinct compositions and characteristics. Plasma comprises platelets, leukocytes, and erythrocytes, whereas serum is obtained after clotting and contains proteins such as albumin, immunoglobulins, and fibrinogen [67,68]. Blood samples provide insights into systemic changes within the body and offer a minimally invasive approach to obtain miRNAs for glaucoma diagnosis and monitoring [7,21]. Stability is a notable feature of blood samples derived from the plasma or serum. However, stability can be influenced by factors such as hemolysis, coagulation, anticoagulant use, sample handling, storage conditions, extraction protocols, and quantification techniques. To minimize the impact of these factors, sterile tubes with or without anticoagulants should be used for blood collection depending on the desired fraction. Prompt centrifugation and the separation of plasma or serum are crucial to prevent hemolysis or coagulation [69,70,71,72,73].

#### 4.1.3. Storage, Extraction, and Quantification of miRNAs in Glaucoma Research

Proper storage and preservation of biological samples obtained from glaucoma patients are essential to ensure the integrity of RNA molecules for miRNA analysis. It is highly recommended that these samples be stored at ultra-low temperatures of −80 °C or below until further processing. This stringent storage condition helps prevent RNA degradation and maintains the stability of miRNAs, enabling accurate and reliable analyses [74]. To extract miRNAs from preserved samples, researchers should employ extraction methods that have been specifically optimized for low-input RNA samples, such as those derived from biological fluids [75]. The utilization of commercial kits or customized protocols designed for miRNA extraction ensures high yields and preserves the quality of the extracted molecules. These optimized methods effectively isolate miRNAs while minimizing contamination and degradation, thereby enabling downstream analysis with confidence [76]. The quantification of miRNAs is a critical step in miRNA analysis. Researchers can choose from various techniques based on their specific research objectives and available resources.

Next-generation sequencing (NGS) is a powerful method that provides a comprehensive profile of miRNA expression, allowing for the identification of novel miRNAs and the detection of low-abundance species [76,77]. Alternatively, quantitative PCR (qPCR) offers high sensitivity and specificity, making it a reliable choice for quantifying specific miRNAs of interest [78,79]. By employing these sensitive and specific techniques, accurate quantitative measurements of miRNA expression can be obtained. Throughout the process of collecting and processing biological samples for miRNA analysis, it is crucial to exercise caution and follow standardized protocols. Properly handling samples, adhering to established procedures, and implementing rigorous quality control measures are paramount to minimize variability and interference in miRNA analysis. By ensuring consistency and reproducibility, researchers can confidently interpret and compare the miRNA data from various experiments and studies.

#### 4.1.4. miRNA Expression Patterns in Glaucoma

miRNAs demonstrate tissue-specific expression patterns, thereby enabling differentiation between glaucoma and control cohorts. For example, Hindle et al. [21] conducted a study that identified 20 circulating miRNAs that were significantly upregulated in patients with glaucoma or exfoliation syndrome (XFS) compared with controls across two ethnic cohorts (Caucasian and Japanese). Among these miRNAs, a combination of miR-637, miR-1306-5p, and miR-3159 demonstrated an excellent ability (AUC = 0.91) to classify glaucoma patients and control subjects with high sensitivity (85%) and specificity (87.5%). In a study by Seong et al. [36], eight significantly upregulated miRNAs were identified in AH samples from patients with NTG compared to controls. These miRNAs, including hsa-let-7a-5p, hsa-let-7c-5p, hsa-let-7f-5p, hsa-miR-192-5p, hsa-miR-10a-5p, hsa-miR-10b-5p, hsa-miR-375, and hsa-miR-143-3p, were predicted to be associated with biological processes such as apoptosis, autophagy, neurogenesis, and aging, which are relevant to glaucoma pathophysiology. The identification of these specific miRNAs provides insights into the molecular mechanisms underlying NTG and potential therapeutic targets. Moreover, several studies have explored the miRNA expression profiles in different ocular tissues, shedding light on the molecular basis of various glaucoma subtypes. Drewry et al. [80] identified differentially expressed miRNAs in AH samples from patients with POAG, exfoliated glaucoma (XFG), and controls. They observed significant alterations in miRNA expression between POAG and controls (e.g., miR-125b-5p, miR-302d-3p, and miR-451a), XFG and controls (e.g., miR-122-5p, miR-3144-3p, miR-320a, miR-320e, and miR-630), and POAG and XFG (e.g., miR-302d-3p). Pathway analysis highlighted the involvement of these miRNAs in glaucoma-related pathways such as focal adhesion, tight junctions, and TGF-β signaling. Furthermore, specific target genes of differentially expressed miRNAs, such as OPTN, TMCO1, and TGF-β1, have been implicated in glaucoma pathogenesis. Rao et al. [81] observed elevated levels of hsa-miR-122-5p, hsa-miR-124-3p, and hsa-miR-424-5p in pseudoexfoliation glaucoma (PXG), influencing specific pathways such as TGF-β1, fibrosis/ECM, and proteoglycan metabolism. These miRNAs target genes involved in the pathophysiology of glaucoma, such as SMAD3/2. Similarly, Czop et al. [82] examined miRNA expression in the AH of patients with pseudoexfoliative glaucoma (PEXG) through using microarrays. They identified twenty differentially expressed miRNAs associated with PEXG, including ten downregulated (e.g., hsa-miR-95-5p) and ten upregulated (e.g., hsa-miR-202-3p) miRNAs. These dysregulated miRNAs may regulate processes such as extracellular matrix imbalance, apoptosis (possibly affecting RGCs), autophagy, and elevated calcium levels. Further studies are required to elucidate the molecular basis of PEXG activity.

In another study, Zhai et al. [83] identified 40 differentially expressed miRNAs (24 upregulated and 16 downregulated) between the control and POAG groups. The upregulated miRNAs included hsa-miR-206, whereas the downregulated miRNAs included hsa-miR-34c-5p and hsa-miR-184. These miRNAs target validated and predicted genes, including BCL2, JUN, and CCND1 and certain long non-coding RNAs (lncRNAs) such as MALAT1 and DCP1A. Several target genes of differentially expressed miRNAs were associated with POAG. The dysregulation of these miRNAs in POAG was confirmed via RT-qPCR, providing further evidence of their potential as biomarkers for glaucoma. Additionally, Cho et al. [40] conducted a study to identify differentially expressed miRNAs in pseudoexfoliation (PEX) glaucoma and NTG. They found upregulated miRNAs (hsa-miR-30d-5p and hsa-miR-320a) and downregulated miRNAs (hsa-miR-3156-5p, hsa-miR-4458, hsa-miR-6717-5p, hsa-miR-6728-5p, hsa-miR-6834-5p, hsa-miR-6864-5p, hsa-miR-6879-5p, hsa-miR-877-3p, hsa-miR-548e-3p, and hsa-miR-6777-5p) in patients with PEX glaucoma. Furthermore, they identified specific pathways associated with PEX glaucoma, such as the TGF-beta signaling pathway. Raga-Cervera et al. [47] compared the expression profiles of eight miRNAs in the tears of individuals with ocular hypertension (OHT) and POAG groups. They identified the differential expression of the following miRNAs: miR-26b-5p, miR-152-3p, miR-30e-5p, miR-125b-2-5p, miR-224-5p, miR-151a-3p, miR-1307-3p, and miR-27a-3p. This study highlights the potential of miRNAs as non-invasive biomarkers for glaucoma detection.

This underscores the crucial role of miRNA stability and specificity in glaucoma research. To ensure accurate detection and comprehensive analysis, it is imperative that miRNAs demonstrate stability across diverse biofluids, including blood, tears, and the AH. This stability guarantees the reliable quantification of miRNA levels and establishes the foundation for their potential use as diagnostic and prognostic biomarkers. Moreover, miRNAs must exhibit specificity for patients with glaucoma. This specificity plays a pivotal role in identifying distinctively dysregulated miRNAs in glaucoma, thereby potentially unveiling miRNA signatures that can serve as invaluable tools for the early detection and continuous monitoring of the disease. By harnessing the unique expression profiles of these specific miRNAs, researchers can develop innovative strategies to improve the accuracy and effectiveness of glaucoma diagnosis, prognosis, and personalized treatment approaches.

### 4.2. Insights into Molecular Pathways and Mechanisms

The investigation of miRNAs in the context of glaucoma provides valuable insights into the intricate molecular pathways and underlying mechanisms associated with the disease [35]. MiRNAs serve as post-transcriptional regulators and exert their regulatory effects by binding to the mRNA transcripts of target genes [5]. By analyzing the expression patterns of miRNAs, researchers can identify dysregulated miRNAs and their associated target genes, thereby unraveling the biological processes implicated in glaucoma [3]. This knowledge not only enhances our understanding of the disease but also facilitates the identification of potential therapeutic targets for the development of novel treatment strategies [84]. The goal of these strategies is to modulate aberrant miRNA-mediated pathways, thereby offering new avenues for therapeutic intervention in glaucoma [3].

Apoptosis and inflammation are molecular pathways regulated by differentially expressed miRNAs in glaucoma [51]. This pathway involves the death and inflammation of RGCs, contributing to optic nerve damage and vision loss. Upregulated miRNAs in the AH of glaucoma patients, including miR-21 [85], miR-146a [86], miR-155 [87], miR-181a [88], miR-181b [21], miR-181c [48], and miR-223 [89], may target genes involved in apoptosis and inflammation, such as PTEN, BCL2, CASP3, IL6, IL8, TNF, and NF-κB [21,48,85,86,87,88,89].

Other processes regulated by differentially expressed miRNAs in glaucoma include extracellular matrix remodeling and autophagy [90]. These involve the degradation and renewal of the extracellular matrix and cellular components of the TM, affecting outflow resistance and IOP [49]. The miRNAs upregulated in the AH of patients with NTG include let-7a-5p, let-7c-5p, let-7f-5p, miR-192-5p, miR-10a-5p, miR-10b-5p, miR-375, and miR-143-3p [36].

Furthermore, focal adhesion, tight junctions, and TGF-β signaling represent another pathway regulated by differentially expressed miRNAs in glaucoma. This pathway involves interactions between TM cells, their surrounding matrixes, and other cells. The upregulated miRNAs in the AH of patients with exfoliated glaucoma include miR-122-5p, miR-3144-3p, miR-320a, miR-320e, and miR-630 [80].

Additionally, oxidative stress and mitochondrial dysfunction constitute molecular pathways that are enhanced in glaucoma, leading to the accumulation of reactive oxygen species and the impairment of mitochondrial function and causing cellular damage and death. MiRNAs such as miR-7, miR-24, miR-27a, miR-29b, and miR-4295 exert antioxidant effects in TM cells, protecting against oxidative stress. They target various genes and pathways, including mTOR, MEK/ERK, and TGFβ1 processing, contributing to the prevention of oxidative damage in glaucoma [86].

The fifth molecular pathway regulated by differentially expressed miRNAs in glaucoma is the Wnt signaling and neuroprotective pathway. This pathway involves the activation of Wnt signaling and the expression of neurotrophic factors in RGCs. However, this pathway is suppressed in glaucoma, which leads to reduced cell survival and function [91]. Furthermore, the Wnt signaling and neuroprotective pathways regulated by miRNAs play a role in cell survival and function in glaucoma. miRNAs such as miR-29b, miR-21, miR-638, miR-100, and miR-125 modulate this pathways by targeting the key genes involved in Wnt signaling regulation, impacting cell survival, fibrosis, inflammation, cell proliferation, migration, and differentiation (e.g., miR-29b targets TCF7L2, BCL9L, SNAI1, and COL3A1; miR-21 targets DKK2, TGFbR2, and Wnt1; miR-100 targets DKK1 and ZNRF3; miR-125 targets DKK3, ZNRF3, RNF43, and APC2) [91].

The elucidation of specific interactions between miRNAs and their target genes has immense potential to deepen our understanding of the intricate molecular mechanisms underlying glaucoma pathogenesis [3,46]. This understanding forms the foundation for the development of targeted therapeutic modalities that aim to reinstate normal miRNA expression or modulate the activity of specific miRNAs [92]. These interventions can potentially restore cellular homeostasis and impede disease progression [92]. Moreover, the functional characterization of dysregulated miRNAs and their target genes offers invaluable insights into the distinct pathophysiological processes of various glaucoma subtypes [21,36,48,85,86,87,88,89,90,91]. By conducting comparative analyses of miRNA expression profiles across these subtypes, researchers can discern subtype-specific miRNA signatures [3]. Such knowledge facilitates the adoption of a more personalized and tailored approach to treatment and management strategies, as interventions can be precisely directed based on the unique molecular profiles of individual patients.

### 4.3. Personalized Medicine

The integration of miRNA research into glaucoma studies has the potential to revolutionize personalized medicine [7]. Traditional glaucoma management often employs a generalized approach that fails to consider the unique characteristics of individual patients, resulting in suboptimal treatment outcomes [93]. However, miRNA profiling provides a more individualized approach by identifying patient-specific miRNA signatures that can inform disease prognosis, predict treatment responses, and guide therapeutic decision-making [40,94].

The integration of miRNA expression profiles with traditional clinical parameters represents a comprehensive and precise diagnostic approach for glaucoma [7,21,81]. While conventional diagnostic modalities, including IOP, optic nerve head assessment, visual field testing, and optical coherence tomography (OCT), are commonly employed in glaucoma diagnosis and management, they possess inherent limitations in terms of sensitivity, specificity, variability, and reproducibility [95,96,97]. MiRNA analysis serves as a valuable complement to these parameters by offering molecular insights into the underlying pathophysiological processes associated with glaucoma [92,98]. Elucidating the dysregulation of miRNA expression patterns will enhance the sensitivity and specificity of glaucoma diagnoses [99]. Notably, the miR-19a/PTEN axis has been implicated in regulating the decline in the axon regenerative capacity of RGCs during development [100]. In addition, the inhibition of miR-194 and miR-664-2 shows significant neuroprotection in RGCs, whereas mimicking miR-181a and miR-181d-5p promotes neuritogenesis [101]. These findings suggest that these miRNAs could aid in patient stratification based on risk factors and disease subtypes and in monitoring treatment responses. Furthermore, the integration of miRNA expression profiles with other clinical parameters, such as genetic variants, demographic factors, and environmental influences, enables the identification of distinct miRNA patterns associated with disease severity, progression rates, and treatment responses [95,96,97]. These miRNA signatures serve as valuable tools to predict disease outcomes, select optimal treatment strategies, and monitor treatment efficacy [102]. Furthermore, the integration of miRNA analysis with diagnostic modalities provides not only a comprehensive understanding of glaucoma but also potential prognostic information. Monitoring changes in miRNA expression over time allows clinicians to assess disease progression and predict treatment responses. This personalized approach to glaucoma management holds significant promise for tailoring therapeutic interventions to individual patients, optimizing treatment outcomes, and minimizing the risk of irreversible vision loss [3,21,102].

Treatment resistance is common in the management of glaucoma, with a significant number of patients demonstrating inadequate responses to standard therapeutic interventions such as topical medications, laser trabeculoplasty, or trabeculectomy [103]. The involvement of miRNAs in the mechanisms underlying treatment resistance stems from their ability to modulate the expression of drug transporters, receptors, metabolizing enzymes, and signaling pathways [3]. The utilization of miRNA-based profiles may provide valuable insights into the mechanisms underlying treatment resistance, thereby facilitating the identification of potential alternative therapeutic strategies. By understanding the miRNA-mediated mechanisms responsible for drug resistance or non-response, clinicians can personalize treatment plans to overcome these challenges and optimize therapeutic outcomes [104]. This approach, rooted in miRNA research, has substantial potential to enhance patient outcomes, reduce treatment expenses, and alleviate the burden of glaucoma on both individuals and healthcare systems.

The integration of miRNA studies in glaucoma with traditional clinical parameters and genetic changes enhances diagnostic accuracy and provides valuable molecular insights into the disease [7,21,81]. By combining miRNA analysis with established clinical parameters such as IOP, optic nerve assessment, visual field testing, and OCT, a more comprehensive understanding of glaucoma pathogenesis and progression can be achieved [95,96,97]. Furthermore, miRNA signatures have shown promise in the identification of treatment resistance in glaucoma. Specific miRNA expression patterns are associated with poor responses to conventional therapies, indicating their potential as predictive markers of treatment outcomes [104]. The identification of miRNA signatures associated with treatment resistance can aid personalized treatment approaches and the development of targeted therapies for individuals who are less responsive to standard glaucoma treatments.

### 4.4. Therapeutic Potential

The therapeutic potential of microRNAs (miRNAs) holds tremendous promise for the development of novel therapeutic strategies in glaucoma. MicroRNAs (miRNAs) have been identified as key regulators of gene expression that influence various biological processes [105]. The dysregulation of miRNA expression has been implicated in the pathogenesis of glaucoma, including retinal ganglion cell apoptosis, neuroinflammation, oxidative stress, and fibrosis [105,106]. Harnessing the therapeutic potential of miRNAs offers a targeted approach to modulate disease-related gene expression and restore normal cellular processes.

One avenue for miRNA therapy in glaucoma is the use of miRNA mimics or agonists. These molecules are designed to restore or enhance the expression of specific miRNAs that are downregulated in glaucomatous tissues. By reintroducing these miRNAs, the expression of target genes associated with disease progression can be suppressed. For example, miR-21, which is downregulated in glaucoma, regulates apoptotic pathways and reduces retinal ganglion cell death [86]. The delivery of miR-21 mimics could potentially mitigate neuronal loss and preserve visual function in patients with glaucoma. Similarly, miR-29b, another downregulated miRNA in glaucoma, modulates extracellular matrix remodeling and fibrosis. Introducing miR-29b mimics may help prevent excessive fibrotic tissue formation and improve tissue homeostasis in the ocular environment [107].

In contrast, miRNA inhibitors or antagonists can be used to block the activity of specific miRNA overexpressed in glaucoma. By inhibiting these miRNAs, it is possible to depress the expression of target genes that are involved in neuroprotective or anti-inflammatory pathways. For instance, miR-155, an miRNA that is upregulated in glaucoma, is associated with increased neuroinflammation. The inhibition of miR-155 activity may alleviate neuroinflammatory responses and promote neuroprotection in glaucoma [108]. Additionally, miR-132, which is upregulated in glaucoma and is involved in oxidative stress pathways, can be targeted with inhibitors to restore redox balance and reduce oxidative damage in the retina [109].

Furthermore, the development of nanocarrier-based delivery systems allows for the efficient and targeted delivery of miRNA therapeutics to ocular tissues affected by glaucoma. These nanocarriers can protect miRNAs from degradation, facilitate their cellular uptake, and enhance their bioavailability at the desired site of action [110]. Targeted delivery systems enable the precise localization of miRNAs in RGCs or other relevant ocular cells, thereby maximizing therapeutic efficacy and minimizing off-target effects. For example, lipid-based nanoparticles loaded with miRNAs have shown successful delivery and gene modulation in ocular tissues, highlighting their potential as miRNA-based glaucoma therapeutics [111,112]. Additionally, hydrogel-based carriers and viral vectors have demonstrated promise in delivering miRNA therapeutics to specific ocular cell types, allowing for tailored treatments [113,114].

In addition to directly targeting miRNAs, another therapeutic approach involves the manipulation of upstream regulators of miRNA expression. Transcription factors or signaling pathways that control miRNA expression can be modulated to restore the balance of miRNA profiles in glaucoma. For example, the transcription factor Nrf2 (nuclear factor erythroid 2-related factor 2) plays a critical role in the oxidative stress response and is involved in the regulation of miRNAs associated with glaucoma pathology. The modulation of Nrf2 activity may restore miRNA homeostasis and provide neuroprotection in glaucoma [115]. Similarly, modulating the Wnt/β-catenin signaling pathway, which has been implicated in glaucoma pathogenesis and miRNA dysregulation, may offer a means to restore normal miRNA expression patterns and mitigate disease progression [116].

Although the field of miRNA therapeutics is still in its early stages, preclinical studies and animal models have shown promising results for glaucoma treatment. For instance, in a rat model of glaucoma, the intravitreal injection of miR-204 mimics reduced retinal ganglion cell loss and improved visual function [117]. However, several challenges must be addressed before miRNA-based therapeutics are translated into clinical practice. These challenges include the development of efficient delivery systems that can traverse ocular barriers, the optimization of target selection to ensure maximum therapeutic impact, and thorough evaluations of safety and efficacy in well-designed clinical trials [110,111,112,113,114].

Nonetheless, the therapeutic potential of miRNAs in glaucoma represents a vast and exciting avenue for future research and has the potential to revolutionize the treatment paradigm for this sight-threatening disease. The ability to modulate gene expression at the post-transcriptional level through miRNA-based interventions offers a precise and targeted approach, potentially leading to improved outcomes, reduced treatment costs, and a reduced burden on individuals and healthcare systems. Continued advancements in miRNA research, delivery systems, and clinical evaluations hold great promise for the development of personalized and effective treatments for patients with glaucoma.

### 4.5. Non-Invasive Monitoring

miRNAs have emerged as highly promising non-invasive tools for monitoring glaucoma, presenting a revolutionary approach for disease management [3,7,51,94,105]. As previously mentioned, traditional monitoring methods for glaucoma, such as IOP measurements, visual field testing, and optic nerve assessment, have limitations in terms of sensitivity, specificity, and their ability to detect early disease progression [118]. In contrast, miRNAs have demonstrated remarkable potential as reliable indicators of glaucoma-associated alterations, offering opportunities to monitor treatment responses [3,92].

Tear fluid, a non-invasive and easily accessible medium, has gained attention as a valuable source for miRNA analysis in glaucoma monitoring. Tears contain a diverse range of miRNAs that can be readily collected using simple techniques such as Schirmer strips or tear collection devices. Several studies have demonstrated altered miRNA expression profiles in the tears of patients with glaucoma compared to those of healthy individuals. For example, miR-146b, a key regulator of the inflammatory response, is upregulated in the tears of patients with glaucoma. Increased levels of miR-146b in tears may reflect ongoing neuroinflammatory processes associated with glaucomatous damage [119]. Similarly, miR-151a-3p and miR-1307-3p have been shown to be downregulated in the tears of glaucoma patients, suggesting their potential role in disease pathogenesis [47]. These findings highlight the potential of tear miRNAs for glaucoma monitoring.

AH offers an additional non-invasive source for miRNA analysis in glaucoma monitoring, as it reflects the molecular changes occurring in the ocular environment and can provide valuable insights into disease progression. Studies have identified differential miRNA expression patterns in the AH of glaucoma patients compared with controls. For instance, miR-29b, a key regulator of extracellular matrix remodeling and fibrosis, has been found to be downregulated in the AH of glaucoma patients [120]. The dysregulation of miR-29b may contribute to the abnormal extracellular matrix deposition observed in glaucoma. Additionally, miR-211, which plays a role in apoptotic pathways, has been shown to be upregulated in the AH of glaucoma patients, suggesting its involvement in retinal ganglion cell death [121]. The analysis of miRNAs in AH provides localized and specific insights into the molecular changes associated with glaucoma.

Moreover, the miRNAs present in peripheral blood circulation offer an additional avenue for the non-invasive monitoring of glaucoma. Blood-based miRNA analysis provides a systemic perspective on glaucoma-related changes, allowing for the identification of miRNA signatures associated with disease progression and treatment response. Numerous studies have demonstrated altered expression levels of specific miRNAs in the blood of patients with glaucoma compared with healthy controls. For example, miR-92a, which is involved in angiogenesis and vascular remodeling, has been found to be upregulated in the blood of glaucoma patients. Elevated miR-210-3p levels may reflect vascular alterations associated with glaucoma progression [122]. Additionally, miR-126, a key regulator of endothelial function [123], has been shown to be upregulated in the blood of glaucoma patients [7]. The dysregulation of miR-126 may contribute to impaired vascular integrity and compromised blood flow in the optic nerve head [86]. These blood-based miRNAs hold promise as non-invasive markers for monitoring glaucoma-related changes throughout the body.

**Figure 3:**
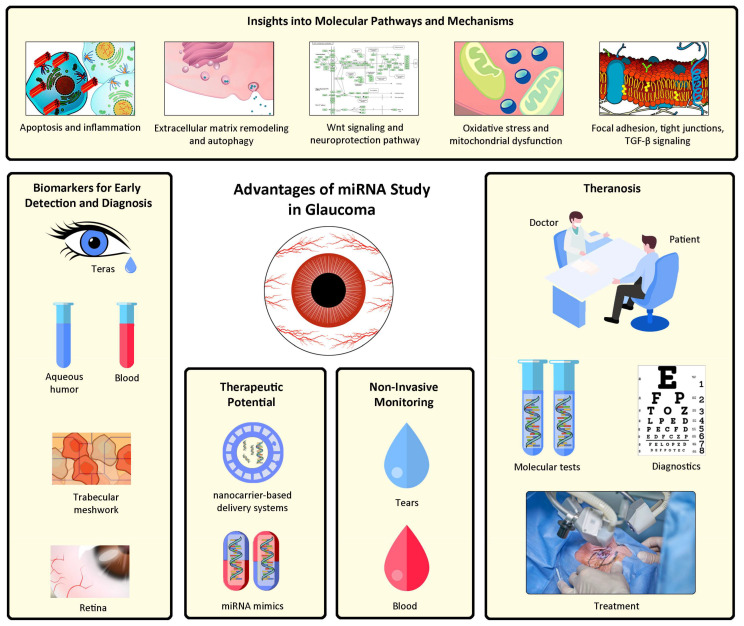
Advantages of miRNA studies in glaucoma.

The non-invasive monitoring of glaucoma using miRNAs offers several advantages over traditional methods. First, miRNA analysis provides early indications of disease progression and treatment response, enabling timely intervention and personalized management strategies. Second, miRNAs can offer insights into the underlying molecular mechanisms of glaucoma, potentially leading to the discovery of novel therapeutic targets. Additionally, the non-invasive nature of miRNA analysis reduces patient discomfort, allows for longitudinal monitoring, and facilitates large-scale population studies. Figure 3 demonstrates the advantages of miRNA analysis in the context of glaucoma.

## 5. Disadvantages of miRNA in Glaucoma

Although miRNA studies in glaucoma hold significant promise, several disadvantages and challenges must be considered. These limitations include variability and lack of standardization; small sample sizes; tissue specificity and accessibility issues; a limited understanding of functional significance, specificity, and specific miRNA targets; and reproducibility and validation concerns.

### 5.1. Variability and Lack of Standardization

One challenge in miRNA research is the lack of standardized protocols for sample collection, storage, and RNA extraction. The variability in these factors can introduce bias and hinder the comparability of results across different studies and laboratories. The standardization of methodologies is crucial to establish consistent miRNA biomarkers or signatures for glaucoma. For example, variations in the isolation method of extracellular vesicles which carry miRNAs can lead to differences in miRNA profiles, affecting the reliability of results. Implementing standardized protocols and quality control measures can address these issues and improve the reproducibility of miRNA studies [18,124].

### 5.2. Small Sample Sizes

Many miRNA studies on glaucoma have used small sample sizes, which may limit the generalizability of the findings. Small cohorts increase the risk of false-positive or false-negative results and may not adequately capture the heterogeneity of patients with glaucoma. Large-scale studies with diverse patient populations are needed to validate miRNA biomarkers and establish their clinical utility. For instance, a study with a small sample size may identify a potential miRNA biomarker, but its true diagnostic or prognostic value can only be confirmed in a larger cohort. Increasing the sample size and conducting multicenter collaborations could enhance the statistical power and reliability of miRNA research on glaucoma [3,7,21].

### 5.3. Samples Specificity and Accessibility

Glaucoma affects multiple biological samples, including the AH, tears, TM, retina, or blood [3]. However, obtaining these samples for miRNA analysis is challenging as invasive procedures such as AH aspiration or retinal biopsies are often required, which limits the feasibility of routine clinical applications and the use of miRNAs as non-invasive biomarkers [125,126]. In addition, obtaining longitudinal samples from the same patient over time is difficult. Alternative sources, such as tears, have been explored for miRNA analysis, but their correlation with specific ocular tissues and relevance in glaucoma remain unclear [7,46]. Developing non-invasive techniques for miRNA profiling, such as analyzing miRNAs in the circulation or tears, could overcome the limitations of tissue accessibility and enhance the clinical utility of miRNA biomarkers.

### 5.4. Limited Understanding of Functional Significance

Although changes in miRNA expression have been observed in glaucoma, the functional significance of these alterations and their precise roles in disease pathogenesis remain largely unknown [43,97,98]. Further research is required to elucidate the specific target genes and pathways regulated by glaucoma-associated miRNAs. Understanding the functional consequences of miRNA dysregulation will provide insight into disease mechanisms and potential therapeutic targets. For example, identifying miRNAs that target genes involved in oxidative stress, neuroinflammation, and retinal ganglion cell apoptosis can help unravel the molecular pathways underlying glaucoma development and progression [47,86,127]. Functional studies such as in vitro experiments or animal models can provide valuable information on the role of specific miRNAs in glaucoma pathophysiology [128,129].

### 5.5. Specificity and Specific miRNA Targets

MiRNAs regulate multiple target genes, and a single miRNA can target several genes involved in different pathways. This lack of specificity makes it challenging to determine the exact contribution of individual miRNAs to glaucoma pathophysiology [130]. Identifying specific miRNA targets and their downstream effects is crucial for understanding the impact of miRNA dysregulation on glaucoma and for the development of targeted therapies. For instance, miR-21 has been implicated in glaucoma and has been shown to target genes involved in cell survival, extracellular matrix remodeling, and inflammation. Investigating the functional consequences of miR-21 dysregulation and its specific target genes may provide insight into the role in glaucoma pathogenesis [92,107,131].

### 5.6. Reproducibility and Validation

**Figure 4:**
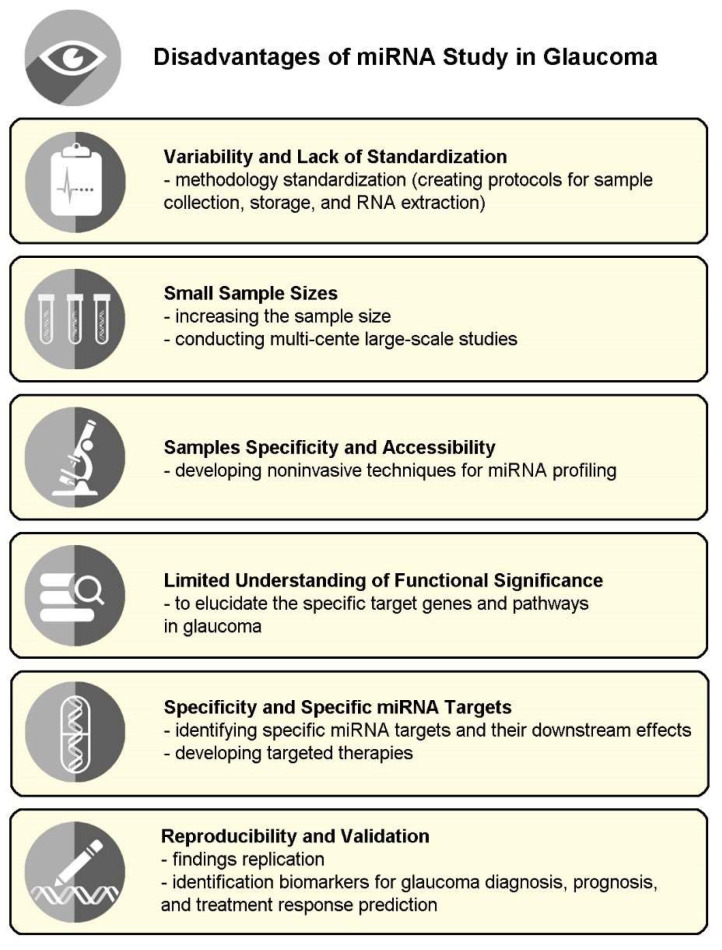
Disadvantages of miRNA studies in glaucoma.

Reproducibility is an important aspect of scientific research [132,133]. Although initial studies have reported promising findings regarding miRNA alterations in glaucoma, the reproducibility of these results across different cohorts and populations needs to be confirmed. The independent validation of miRNA biomarkers in larger and more diverse patient cohorts is necessary to establish their clinical relevance, reliability, and robustness as diagnostic and prognostic tools. The replication of findings from multiple studies strengthens the evidence and increases confidence in the utility of miRNA biomarkers for glaucoma diagnosis and prognosis and the prediction of treatment responses. Collaborative efforts among researchers and the establishment of miRNA consortia can facilitate the replication and validation of miRNA findings. Figure 4 illustrates the limitations of miRNA studies in glaucoma.

## 6. miRNA Studies in Glaucoma: Prospects and Limitations

In summary, the study of miRNAs in glaucoma has both advantages and disadvantages. MiRNA research holds immense potential to revolutionize glaucoma management by offering personalized and targeted approaches for diagnosis, prognosis, and treatment. These unique miRNA signatures offer valuable insights into disease severity, progression rates, and treatment responses, enabling clinicians to make well-informed decisions regarding patient care. Additionally, miRNAs serve as non-invasive biomarkers, facilitating easier and less invasive disease progression and treatment efficacy monitoring.

Nevertheless, several challenges and limitations are associated with miRNA studies in glaucoma. The variability and lack of standardization in sample collection and analysis methods introduces bias and hinders the comparability of results across studies. Small sample sizes and tissue specificity issues limit the generalizability of the findings and accessibility of relevant ocular tissues for analysis. The functional significance of miRNA dysregulation and its specific targets in glaucoma pathogenesis remain incompletely understood, necessitating further research to elucidate these mechanisms. Furthermore, the non-specific targeting of miRNAs to multiple genes and pathways poses challenges in determining their precise contribution to the pathophysiology of glaucoma.

Addressing these challenges requires persistent research efforts, including method standardization, large sample sizes, and the functional characterization of miRNAs. Collaborative studies and validation in independent cohorts are critical to establish the clinical relevance and reliability of miRNA biomarkers for glaucoma. By addressing these limitations, we can enhance our understanding of the involvement of miRNAs in glaucoma and pave the way for personalized medical approaches that optimize patient outcomes and minimize the burden of glaucoma on individuals and healthcare systems.

## 7. Bioinformatic Pipeline and Data Governance

An important aspect of experimental research is the creation of opportunities to combine omics data and clinical information. The best way to present them is through a structured compilation of high-quality NGS data, together with reliable clinical features.

A critical element of the approach for creating large-scale databases is the proper preparation (preprocessing) of raw data generated from NGS analysis. The subsequent stages of the bioinformatics processing path must be provided with a number of control points to ensure the appropriately high quality of the obtained sequences.

### 7.1. Bioinformatic Pipeline

**Figure 5:**
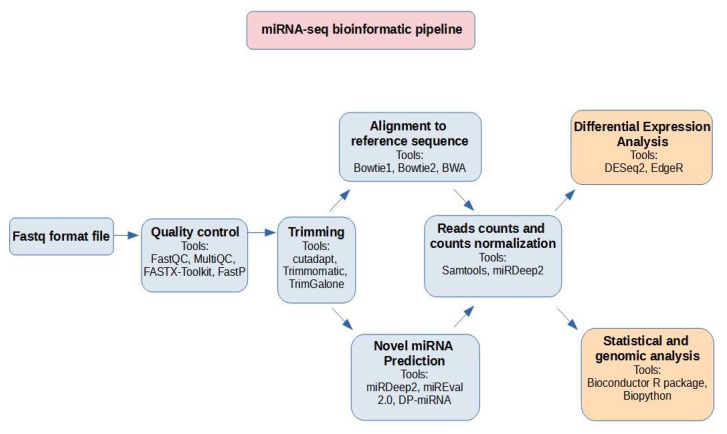
Typical miRNA sequencing bioinformatic pipeline scheme.

RNA-seq analysis based on NGS data has become the standard to analyze gene expression at the whole transcriptome level in various diseases, including eye disorders. The advent of RNA-seq analysis has facilitated genome-wide expression profiling, including the identification of novel and rare transcripts such as non-coding RNAs and novel alternative splicing isoforms. One branch of RNA-seq analysis involves miRNA sequencing [134]. The creation and processing of both genetic and clinical data are often based on different methodologies that complicate their storage and utilization. The data generated by miRNA-seq experiments are complex and voluminous, requiring the development of sophisticated bioinformatics pipelines to process, analyze, and interpret information effectively and reproducibly [135]. The miRNA-seq bioinformatics pipeline is a structured sequence of computational tools and algorithms designed to handle various stages of data analysis (Figure 5) [136].

Raw output data received from NGS platforms such as Illumina or Oxford Nanopore can be in FASTQ format files that contain nucleotide sequences and their corresponding quality scores. Various pipelines for miRNA-seq have been created; however, typical raw data preprocessing begins with read quality control and trimming [77,137,138] to remove adaptors and low-quality bases and verify sequence quality [139]. Generally, reads that are either too short or long are excluded. Next, the clean reads are mapped to mature reference miRNA sequences. The most used reference sources for miRNA alignment are the miRBase database [140] and the whole genome [141]. Aligners such as Bowtie1, Bowtie2, and BWA are used because of their ability to map short transcripts (such as miRNAs) to large genomes [77,134,137,141]. The next step is to count the mapped reads from the SAM/BAM [140] format files or output files from the package script (e.g., miRDeep2) [138]. The read counts are normalized and used for differential expression and other statistical analyses. The second path, where row unmapped reads can be used, is novel miRNA prediction [142]. The computational prediction of miRNAs is based on homologous searches, comparative genomics, and machine learning (ML). The first two methods can accurately identify miRNAs; however, they cannot identify non-homologous or species-specific miRNAs because they mainly depend on sequence conservation [142]. The proposed tools used in the bioinformatics pipeline for miRNA sequencing are shown in Table 1.

### 7.2. Data Governance

To support the diagnostic process, it is necessary to manage patient data effectively. This applies to the proper design of both the database architecture and specification of data processing. As the diagnostic process increasingly uses information from different data sources (clinical data are combined with imaging and genetic data), proper standardization of the data management process is crucial.

**Figure 6:**
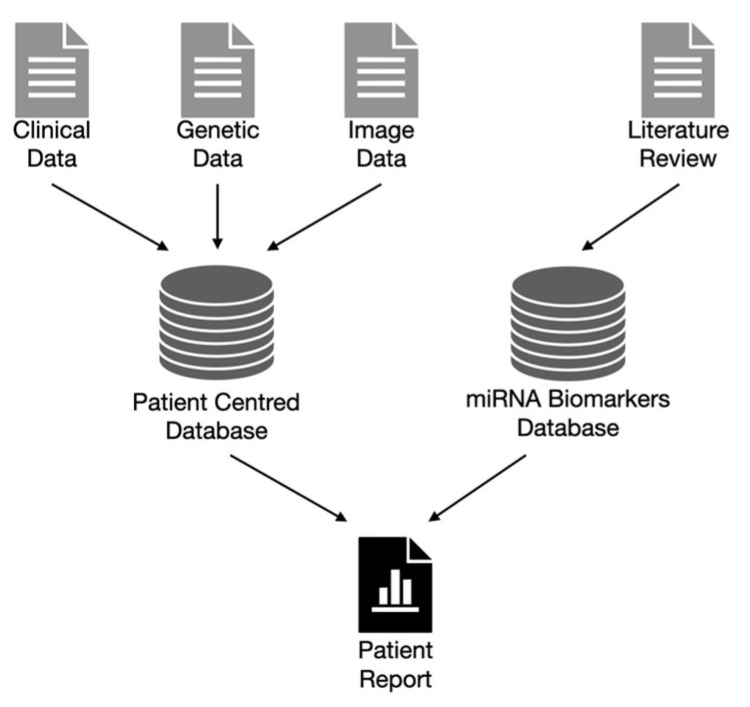
Integration of patient-centered database with generic biomarker databases. A patient database aggregates a variety of patient data and allows for associations between different characteristics and modalities to be determined (e.g., genetics with imaging). The biomarker database aggregates data on biomarkers identified in the literature and with prognostic signatures.

The following proposition is based on the findable, accessible, interoperable, and reusable (FAIR) principles [143], which, if properly implemented, increase the efficiency of using the available data. FAIR refers to the ability, accessibility, interoperability, and reusability of digital assets. The ability principle requires that the stored information be properly described by rich metadata fields. To satisfy the accessibility principle, it is crucial to use standardized API protocols and relational databases. Because data are generated at different points in the process, the interoperability principle requires these data sources to be linked (e.g., shared identifiers). No less important is the reuse principle, which requires the design of databases to consider various future scenarios for the use of specific resources. To meet these principles for glaucoma data analysis involving miRNAs, it is important to combine two data hubs: a database that stores patient data and databases that store genetic biomarker data (Figure 6).

The patient database includes information collected during patient examinations, such as clinical and diagnostic data. However, reports regarding other projects, such as DECODE-EYE [144], indicate that patient data often require additional integration with the available registries. Therefore, it is necessary to include them in the database identifiers that allow for such integration. Data from registries can cover an entire population with specific diseases, and such registries can contain a few hundred to more than a million records (for example, this registry contains data on 17.3 million people [145]).

Studies featuring the use of the EPISAFE project [146] have shown that patient data not only consist of clinical characteristics collected during the first interview but also time-dependent characteristics collected at subsequent visits. Therefore, patient databases should be able to supplement patient data with new information collected during the treatment process. Patient data are usually stored in relational databases such as MySQL or PostGress. This includes processed patient genetic information such as mutations or variants of specific genes or miRNAs. Raw genetic data can be much larger in volume; therefore, cloud solutions [147] are often used to store these.

To monitor the presence of disease-related genetic signatures in a patient, a second database that stores information on known disease markers is necessary. One of the most versatile databases is EyeDieseases [148], which aggregates information collected on gene expression, mutation, or miRNA levels. This database integrates genetic, transcriptomic, and epigenetic data.

In addition to these universal databases, specialized databases containing miRNA-based prognostic signatures are available. An example of such a database is EBD, which contains 35 miRNA-based predictive signatures [149], and miRNeye, which contains 285 predictive signatures but focuses on eye diseases in mice [150]. Information on gene expression in relation to eye diseases is also available. These expression data are available from iSyTE [151] and GluDB [152].

Glaucoma analysis often involves the use of genetic data and fundus photographs. Such data can also be used to automatically extract the specific features observed in a photo. One of the largest databases of photographs of the eye is FIVES [153]. The available images allow for the creation of predictive ML models. Many image databases, such as the Retinal fundus multi-disease image dataset (RFMiD) database, specialize in high-resolution images [154]. Khan et al. [155] have provided a systematic review of the available eye image databases.

## 8. Conclusions

In summary, microRNAs play a pivotal role in regulating gene expression in processes relevant to glaucoma, including apoptosis, inflammation, oxidative stress, and neuroprotection. Their versatile applications encompass biomarker discovery, diagnostics, therapeutics, and non-invasive monitoring, offering the potential for precision in glaucoma management. The landscape of miRNA research in glaucoma involves a spectrum of profiling tools, each with unique attributes. However, this journey is not without challenges, including those pertaining to variability, sample-specific issues, and the integration of bioinformatics. Overcoming these hurdles requires robust protocols, collaborative efforts, and dedicated miRNA databases. MiRNA research has shed light on the molecular foundations of glaucoma, opening doors to innovative diagnostic and therapeutic possibilities. Yet, the path toward implementing miRNA-based treatments demands rigorous scrutiny and validation. In this context, this article introduced a comprehensive bioinformatic pipeline tailored for miRNA-seq data analysis. This pipeline covers quality control, trimming, alignment, novel miRNA prediction, and differential expression analysis using notable tools like FastQC, cutadapt, Bowtie2, miRDeep2, and DESeq2, providing a cohesive framework for data interpretation. It is imperative to emphasize the significance of data governance and standardization in amalgamating patient data and genetic biomarker information. Following the principles of Findability, Accessibility, Interoperability, and Reusability (FAIR), we encourage the effective utilization of available data. Additionally, our review of existing databases like EyeDiseases, EBD, miRNeye, iSyTE, GluDB, FIVES, and RFMiD will hopefully help to contextualize the landscape of miRNA-centric research in eye diseases and glaucoma. These databases offer a valuable resource for future research endeavors and collaborations, advancing our understanding and management of glaucoma through miRNA studies.

## Figures and Tables

**Table 1:** Proposed tools used in bioinformatics pipeline for miRNA sequencing.

Pipeline Step	Proposed Tools
Quality control	FastQC, MultiQC, FASTX-Toolkit, FastP
Trimming	Cutadapt, Trimmomatic, TrimGalone
Alignment	Bowtie1, Bowtie2, BWA
Novel miRNA prediction	miRDeep2, miREval 2.0, DP-miRNA
Differential Expression Analysis	DESeq2, EdgeR

## Data Availability

No new data were created or analyzed in this study.
